# Application of recombinase polymerase amplification with lateral flow assay to pathogen point-of-care diagnosis

**DOI:** 10.3389/fcimb.2024.1475922

**Published:** 2024-11-18

**Authors:** Yilian Zhao, Yan Wei, Chao Ye, Jinmeng Cao, Xiaoxing Zhou, Mengru Xie, Jilin Qing, Zhizhong Chen

**Affiliations:** ^1^ The First Clinical Medical College of Guangxi Medical University, Nanning, China; ^2^ Precision Joint Inspection Centre, The People’s Hospital Guangxi Zhuang Autonomous Region and Guangxi Academy of Medical Sciences, Nanning, China; ^3^ Graduate College, Guangxi University of Chinese Medicine, Nanning, China; ^4^ School of Clinical Medicine, Guilin Medical University, Guilin, China; ^5^ Department of Scientific Research, Affiliated Hospital of Chifeng University, Chifeng, China; ^6^ Center for Reproductive Medicine and Genetics, The People’s Hospital of Guangxi Zhuang Autonomous Region and Guangxi Academy of Medical Sciences, Nanning, China

**Keywords:** recombinase polymerase amplification, lateral flow, POCT, pathogen, diagnosis

## Abstract

Since the outbreak of the new coronavirus, point-of-care diagnostics based on nucleic acid testing have become a requirement for the development of pathogen diagnostics, which require the ability to accurately, rapidly, and conveniently detect pathogens. Conventional nucleic acid amplification techniques no longer meet the requirements for pathogen detection in low-resource, low-skill environments because they require specialist equipment, complex operations, and long detection times. Therefore, recombinant polymerase amplification (RPA) is becoming an increasingly important method in today’s nucleic acid detection technology because it can amplify nucleic acids in 20–30 minutes at a constant temperature, greatly reducing the dependence on specialist equipment and technicians. RPA products are primarily detected through methods such as real-time fluorescence, gel electrophoresis, lateral flow assays (LFAs), and other techniques. Among these, LFAs allow for the rapid detection of amplification products within minutes through the visualization of results, offering convenient operation and low cost. Therefore, the combination of RPA with LFA technology has significant advantages and holds broad application prospects in point-of-care (POC) diagnostics, particularly in low-resource settings. Here, we focus on the principles of RPA combined with LFAs, their application to pathogen diagnosis, their main advantages and limitations, and some improvements in the methods.

## 1 Introduction

Pathogens that can cause human infections are mainly bacteria, viruses, fungi, and parasites. The presence of disease-inducing pathogens poses a risk to human life and health. Moreover, these pathogens can spread within populations by numerous means. If left undiagnosed and untreated in a timely manner, they may lead to pandemics (Casadevall, 2017), such as the recent global outbreak of the novel coronavirus. These pandemics not only endanger human life but also exert a significant impact on societal economic development (Cui et al., 2019). Therefore, for pathogenic infections, early and rapid diagnosis is crucial. As a result of these urgent needs, the development of reliable point-of-care testing (POCT) methods has attracted significant attention in recent years, and the World Health Organization’s criteria for POCT are that the test must be affordable, sensitive, specific, user-friendly, fast and robust, require no equipment to conduct, and be deliverable to the end user (Smith et al., 2018). Point-of-care (POC) diagnostics are also known as bedside or additional laboratory tests. They typically provide results within a few hours, eliminating reliance on complex instrumentation and specialized technicians, and are suitable for rapid on-site testing and diagnosis of pathogens in situations where resources are lacking (Kumaran et al., 2023).

Traditional pathogen detection methods, such as polymerase chain reaction(PCR), Enzyme-linked immunosorbent assay (ELISA), pathogen isolation, and culture, require professional technicians and complex instruments, which limits the diagnosis of pathogens to specialized laboratories and fails to meet the demand for rapid and immediate diagnosis and the diagnosis of pathogens in resource-limited areas (Zeng et al., 2024). In recent years, the development of isothermal amplification (INA) technologies, such as rolling circle amplification (RCA), loop-mediated isothermal amplification (LAMP), and recombinase polymerase amplification (RPA), has increased the prospects for the development and application of POC diagnostics (Srivastava and Prasad, 2023). INA technology, which does not require complex thermal cycling instruments but only a heated instrument, can achieve exponential amplification of nucleic acids at a constant temperature, greatly reducing the cost of equipment and time (Kang et al., 2022). Compared with other amplification methods, RPA only requires the design of two primers and can be completed in 30 minutes at 20–42°C (Li et al., 2024). Usually, the amplification products of RPA can be analyzed by agarose gel electrophoresis, fluorescence, and lateral flow assays (LFAs) (Munawar, 2022).

LFA is simple to operate and does not require complex instruments and professional personnel, and the results can be interpreted by visual observation, which allows for rapid diagnosis. It is currently one of the most commonly used strategies for RPA product detection (Liu et al., 2021; Zheng et al., 2023). LFA was first used in urine-based pregnancy tests (Posthuma-Trumpie et al., 2009) for the detection of human chorionic gonadotropin and is now widely used in pathogen diagnosis (Yang et al., 2021), environmental monitoring (Liu et al., 2022), and food safety testing (Yang et al., 2021). It is one of the fastest-growing, most widely used, and highly promising analytical methods in the field of POCT.

Combining RPA with LFA can provide results within 30–60 minutes, and this can be performed without the need for sophisticated instruments and trained personnel (Nan et al., 2023b). For applications outside the laboratory, the combination of RPA and LFA is the best choice, offering advantages of convenience, speed, and low cost, with great applicability in POC diagnostics and lower costs, making it more suitable for the rapid diagnosis of pathogens in resource-poor settings (Reynolds et al., 2023).

## 2 Principle of recombinase polymerase amplification

**Figure 1:**
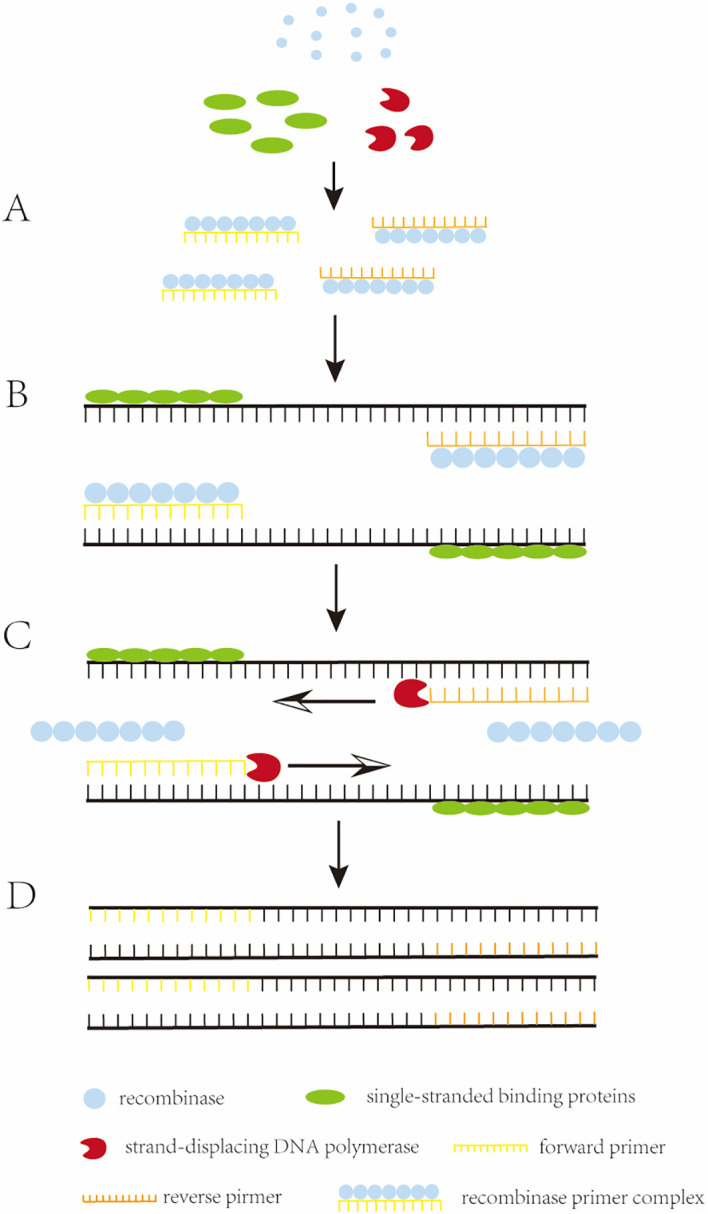
The working principle of RPA. **(A)** Recombinase binds to an oligonucleotide primer to form a recombinase-primer complex, which then searches for homologous sequences in the double-stranded DNA. **(B)** when the primer matches the complementary template sequence, the recombinase catalyzes the binding of the primer to the homologous sequence, and in order to stabilize this structure, a single-stranded binding protein (SSB) binds to the complementary. **(C)** the recombinase dissociates and the 3’ end of the primer is exposed, recognized and extended by strand-substitution DNA polymerase. **(D)** exponential amplification is obtained by the cyclic repetition of this process.

Recombinase polymerase amplification (RPA) is the *in vitro* amplification of target nucleic acids using recombinases and polymerases (Munawar, 2022). It involves a reaction mechanism that relies on a key process of DNA metabolism, a natural cellular adaptation process known as homologous recombination (Li et al., 2019a). The initiation reaction of RPA requires three key proteins (recombinase, single-stranded binding proteins (SSB), and strand-displacing DNA polymerase), which together with auxiliary components (including recombinase-loading factors, crowding agents, energy and fuel components, and salt molecules), among others, perform the reaction (Mota et al., 2022). The reaction begins when the recombinase binds to the oligonucleotide primer with the assistance of the recombinase loading factor to form a recombinase-primer complex. The formed complex searches for homologous sequences in double-stranded DNA, and when the primer matches the complementary template sequence, the recombinase catalyzes the binding of the primer to the homologous sequence to form a D-loop structure. To stabilize this structure, a SSB binds to the complementary untwisted strand to stabilize the D-loop. Subsequently, the recombinase dissociates, and the 3’ end of the primer is exposed. The strand-displacing DNA polymerase then recognizes and extends the primer, and the process is cycled to achieve exponential amplification of nucleic acids. The basic principle of RPA was illustrated in **Figure 1** (Feng et al., 2023).

## 3 Detection of recombinase polymerase amplification products

RPA can be combined with a variety of assays, and it can be monitored by endpoint detection (after amplification) or in real time (during amplification). Different primers and probes can be used depending on the detection strategy, and the common techniques used include agarose gel electrophoresis, LFAs, real-time fluorescence detection, and electrochemical detection. Typically, endpoint assays require less equipment than real-time assays, which reduces the overall cost of the test and may therefore be more suitable for low-resource environments (Lobato and O’Sullivan, 2018).

### 3.1 Agarose gel electrophoresis

**Figure 2:**
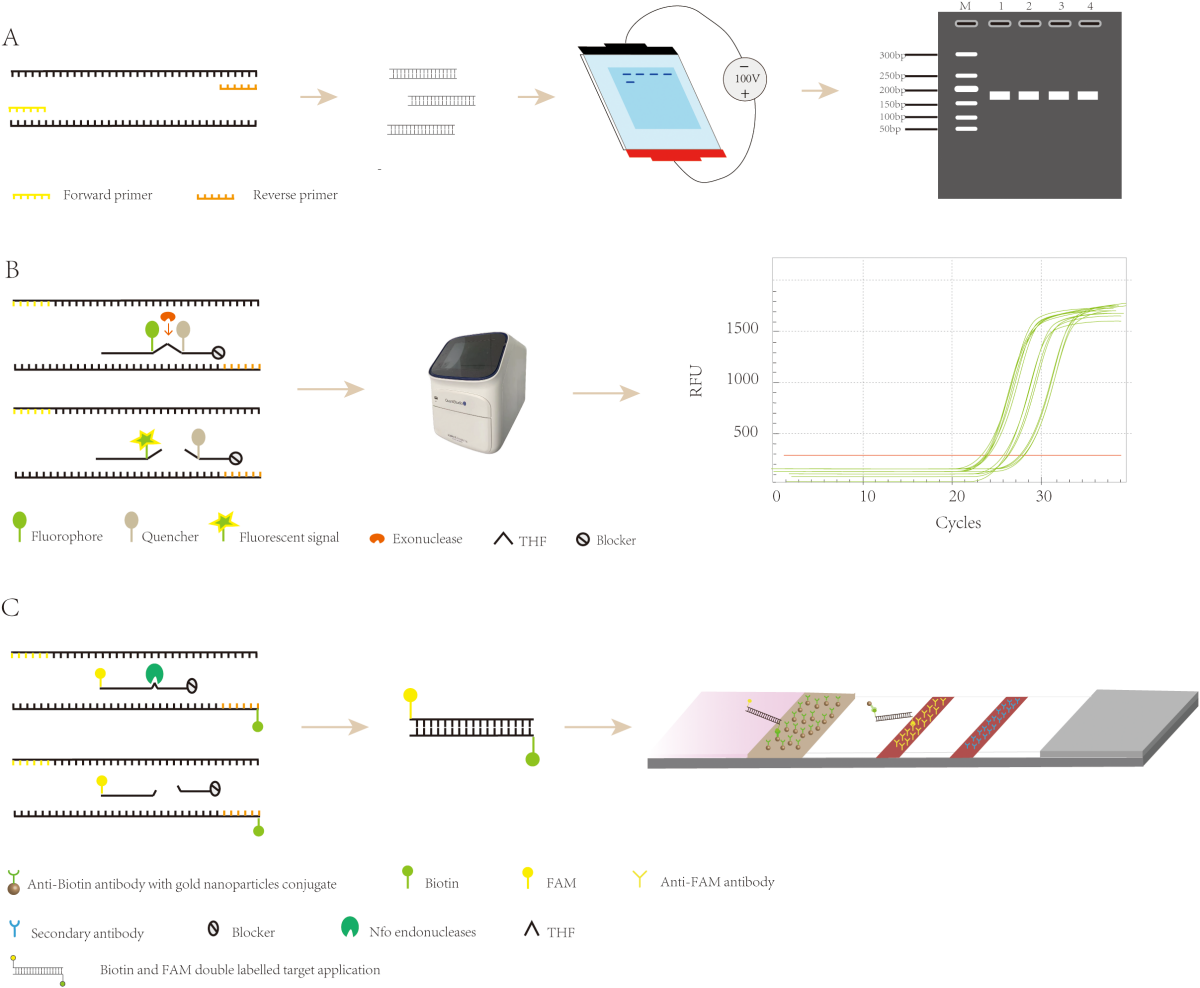
Recombinase polymerase amplification detection strategy. **(A)** Principle of agarose gel electrophoresis detection. **(B)** The exo probes for real-time detection. **(C)** The exo probes for lateral flow detection.

Agarose gel electrophoresis is a method employed for detecting RPA products. This technique requires minimal equipment, namely just a horizontal electrophoresis instrument. Following electrophoresis and UV excitation of the gel, the product can be detected (**Figure 2A**). The cost of this method is low; however, it also has its shortcomings. Prior to detecting the amplification product, it needs to be purified to eliminate interference from other components of the RPA system (such as proteins and aggregating agents) and prevent the occurrence of smeared bands in the electrophoresis results (Lobato and O’Sullivan, 2018). Ahmed et al. (Ahmed, 2015). applied a basic detection system of agarose gel electrophoresis for the detection of Madurella mycetomatis (Mycobacterium maduroides), which minimized the cost of the assay, with a minimum lower limit of detection of 0.23 ng of DNA, a diagnostic sensitivity of 100%, and a specificity of 100%, which is adequate for the detection of Mycobacterium maduroides. However, agarose gel electrophoresis increases the cost and time of the assay due to the requirement for the purification of the amplified product. This disadvantage has led to agarose gel electrophoresis being gradually replaced by other more convenient and rapid assays.

### 3.2 Real-time fluorescence

Real-time fluorescent RPA (RT-RPA) is similar to RT-PCR in that the RT-RPA system consists of a fluorescent motif, a tetrahydrofuran modification site, a quenching motif, and a blocking site (Space C3) located at the 3’ end. These internal modifications are localized to approximately 15 nucleotides at the 3′ end of the probe (Euler et al., 2012a), and when the probe binds to the template, the THF site is recognized and cleaved by the nucleic acid exonuclease, which separates the fluorescent and quenching groups, resulting in the generation of a fluorescent signal, which accumulates in parallel with amplification and can be detected by specialized instruments (**Figure 2B**) (Feng et al., 2023). RT-RPA has been widely used for the detection of viruses, bacteria, parasites, and other pathogens. Zhu et al. (Zhu et al., 2022b) developed a rapid and sensitive real-time RPA-based method for the detection of Vibrio mimicus by targeting the vmh gene and tested it on artificially contaminated samples. The organism could be detected in less than 20 minutes, and the test required less equipment than a real-time fluorescence polymerase chain reaction. Additionally, the RPA method is resistant to inhibitors in plasma samples, which reduces the time required for DNA extraction, and the detection process takes less time. Euler et al. (Euler et al., 2012b) developed a new method that involved performing the test at 42°C for 20 minutes, which included a moving precision fluorescence qualitative real-time RPA based on a small ESEQuant tube scanning device (Qiagen Lake Constance GmbH, Stockach, Germany) for the detection of Tourette’s Rift Valley Fever Virus. The ESEQuant electron tube scanning unit is a fluorescence sensor that collects fluorescence signals over time and records the increase in fluorescence signal in real time. Its detection results are interpreted by combining thresholds and signal slopes for second-order derivative analysis. The method was shown to have the same sensitivity as the real-time fluorescent polymerase chain reaction, but its reaction was much faster, and the ESEQuant tube scanner device has great potential for field use and POC detection due to its small footprint and light weight. However, real-time fluorescent RPA relies on specialized instruments to detect amplification products in real time, which poses a challenge to its application and popularity in low-resource environments.

### 3.3 Lateral flow

**Figure 3:**
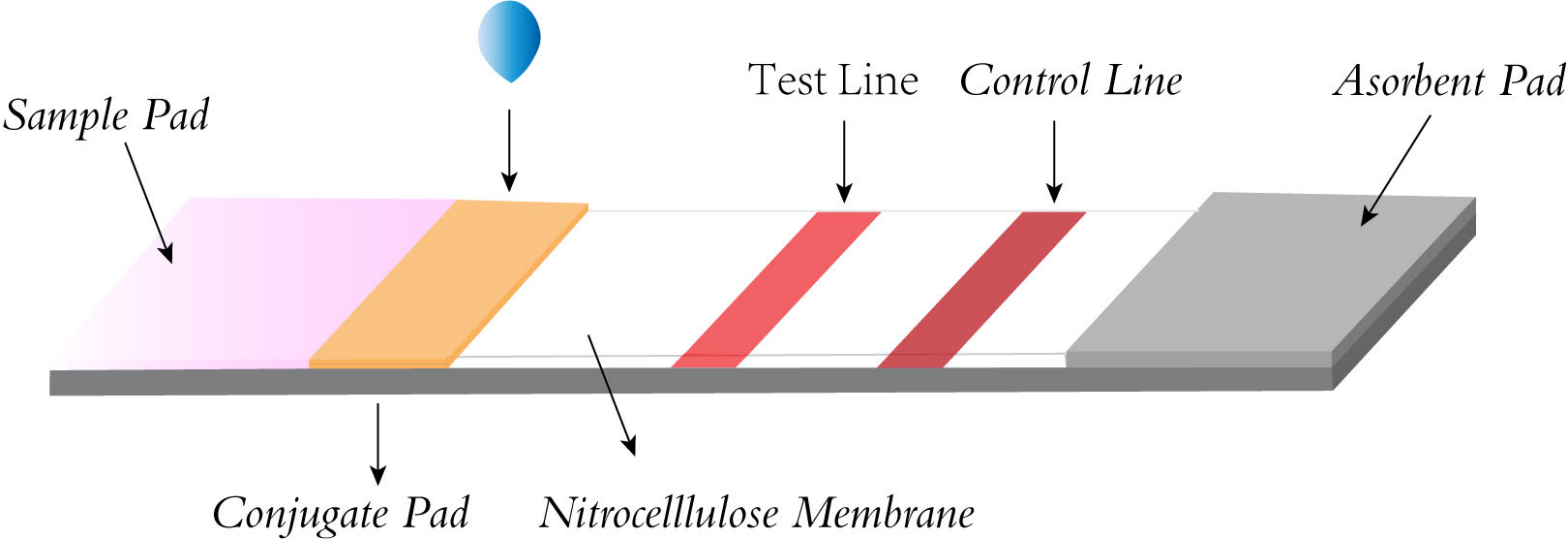
Structural diagram of lateral flow strip.

The LFA primarily involves the use of a sample pad, conjugate pad, nitrocellulose membrane (NC membrane), and absorbent pad, all interconnected with overlapping edges. The sample pad, where the sample is introduced, evenly distributes it onto the conjugate pad, which is impregnated with labeled biosensing molecules, typically colloidal gold particles. Here, the analyte binds to the labeled antibody, forming a complex that travels through the NC membrane’s test and control lines via capillary action. The complex is then captured by a secondary antibody, causing a visible color change due to the accumulation of colloidal gold. This allows for easy visual interpretation of the results. The absorbent pad, located at the strip’s end, enhances capillary flow, soaks up excess fluid, and prevents sample reflux. (**Figure 3**) (Koczula and Gallotta, 2016; Li and Macdonald, 2016; Ince and Sezgintürk, 2022; Nan et al., 2023b). Because LFA has the advantages of being fast, convenient, low cost, and easy to operate, it has been widely used in various fields such as healthcare, food safety, and environmental monitoring (Park, 2022), and can be used to detect proteins (Nan et al., 2023a), nucleic acids (Li et al., 2023b), antigens and antibodies, and semi-antigens (Li et al., 2023a).

**Figure 4:**
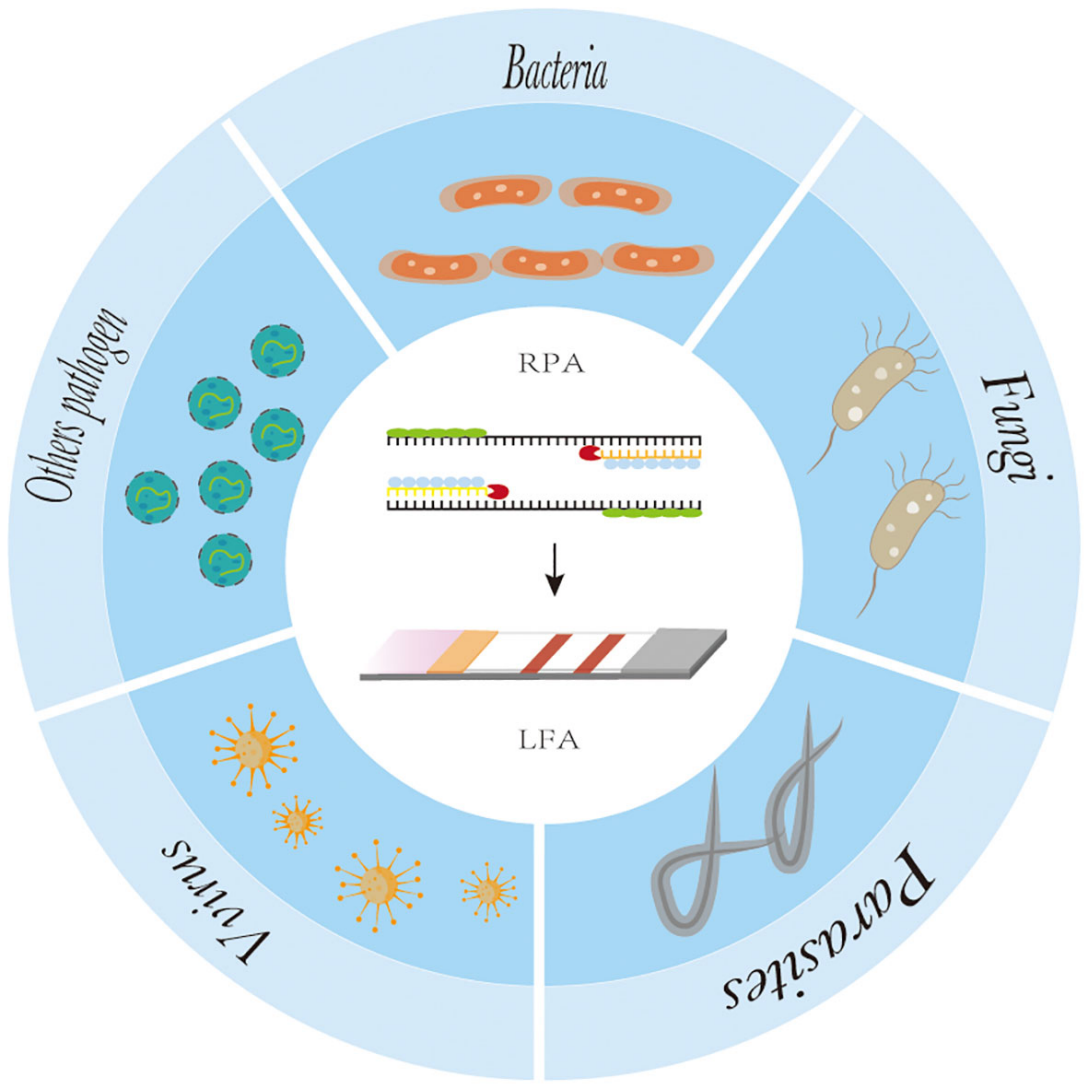
The application of recombinase polymerase amplification with lateral flow assay at pathogen point-of-care-diagnosis.

RPA-LFA is based on the principle of RPA, where the primer is labeled with a biotin marker, the probe is labeled with a fluorescein (FAM/FITC) marker, and the primer and probe undergo an amplification reaction with the target nucleic acid, resulting in an amplified product that carries both the fluorescein and biotin markers. Using the TwistAmp kit (Yao et al., 2024) as an example, a sequence of approximately 46–52 bases is selected according to the principle of complementary pairing; the THF site is labeled approximately 15 bases from the 5’ end; the carboxyfluorescein (FAM) and the blocker are labeled at the 5’ end of the sequence; and biotin is labeled at the 5’ end of the reverse primer. During amplification, when the probe recognizes and binds to the target sequence, the nucleic acid endonuclease cleavage activity is activated, recognizing the THF site and cleaving it. After this, the DNA polymerase extends the probe and primer to produce double-stranded DNA labeled with FAM at one end and biotin at the other. The labeled amplification product is added to the test strip, and because the affinity-labeled colloidal gold nanoparticles (AuNPs) bind to biotin, the amplification product with colloidal AuNPs is produced first as the sample flows through the conjugate pad. As the amplified fragment moves forward with the AuNPs, the labeled fluorescein moiety can bind to the anti-FAM antibody on the test line. Due to the accumulation of AuNPs, the test line turns red (Tan et al., 2022), and the results can be read by visual observation (**Figure 2C**) (Jiang et al., 2019). Lateral flow analysis has the advantages of convenience, rapidity, low cost, and adequate sensitivity and specificity compared to other methods, which makes it more conducive to POC detection (Huang et al., 2021). Nowadays, the combination of RPA and lateral flow analysis systems has been widely used for the detection of pathogens (**Figure 4**).

## 4 Recombinase polymerase combined with lateral flow analysis in pathogen detection

At present, RPA-LFA is being widely used for the detection of pathogens such as viruses (James et al., 2018; Shelite et al., 2021), bacteria (Chen et al., 2023), fungi (Lin et al., 2024), parasites (Crannell et al., 2014; Lei et al.,2022), and other pathogens (Qi et al., 2018), and many studies have proven that they have adequate sensitivity and specificity and greatly shorten the detection time, providing a reliable method for the rapid diagnosis of diseases (**Table 1**).

**Table 1:** The diagnostic performance of RPA-LFA in the detection of different pathogens.

Pathogenic	Target	LoD	Specificity	Assay time	Reference
SARS-CoV-2	RNA	1 copy/ul	100%	30min	(Liu et al., 2021)
influenza viruses	RNA	500 copies/reaction	100%	35min	(Sun et al., 2019)
Coxsackievirus A6	RNA	10 copies/reaction	100%	35min	(Xie et al., 2021)
Hepatitis B virus	DNA	10 copies/reaction	100%	30min	(Zhang et al., 2021)
Klebsiella pneumoniae	DNA	10 copies/uL	100%	30min	(Bhat et al., 2022)
Salmonella spp	DNA	1.1 × 102 f g	100%	25min	(Zhao et al., 2021)
Campylobacter jejuni	DNA	46 CFU/mL	100%	30 min	(Chen et al., 2023)
Cryptococcus spp.	DNA	30 copies/reaction	95.8%	35 min	(Ma et al., 2019)
Candida albicans	DNA	1 CFU/reaction	100%	25 min	(Wang et al., 2021)
Candida krusei	DNA	10 CFU/reaction	100%	20 min	(Zhao et al., 2022)
Trichinella spp.	DNA	10 fg/reaction	100%	25 min	(Li et al., 2019b)
Clonorchis sinensis	DNA	10 fg/reaction	100%	20min	(Ma et al., 2023)
Toxoplasma gondii	DNA	0.1ocyst/reaction	100%	20min	(Wu et al., 2017)
Mycoplasma pneumoniae	DNA	10 copy/ul	100%	25min	(Zhu et al., 2022a)

### 4.1 Viruses

Viruses spread quickly and can cause pandemics of human disease, and severe viral infections can threaten the lives of patients; therefore, there is a great need for the early and rapid detection of viruses. Because the SARS-CoV-2 pandemic caused a global medical crisis, there is an urgent need for a simple and rapid diagnostic test that rapidly detects the virus. Liu et al. (Liu et al., 2021) combined reverse transcription RPA and LFA to establish a method for the rapid and sensitive detection of SARS-CoV-2 and integrated the assay system into a microfluidic chip; the assay requires only one simple nucleic acid extraction and loading, and the results can be obtained after approximately 30 minutes of reaction, without the need for specialized instrumentation and training to enable low-cost screening for COVID-19 in resource-limited areas. Sun et al. (Sun et al., 2019) designed a dual RPA with LFAs for the simultaneous detection and identification of influenza A and B viruses, enabling the identification of influenza virus infections and differentiating them from other respiratory infections. After optimizing the parameters of the method, such as reaction temperature, reaction time, and primer and probe concentrations, the method has high specificity and sensitivity, with a lower limit of detection of 50 and 500 copies for influenza B and influenza A viruses, respectively, and no cross-reactivity with other pathogens. This rapid and simple test allows for rapid diagnosis of viral infections at an early stage, effectively stopping the spread of common respiratory pathogens. Early diagnosis and targeted treatment also reduce the severity of the disease and mortality rates and improve the level of global healthcare coverage. Hand, foot, and mouth disease (HFMD) is a common infectious disease that occurs in children, affecting millions of children each year, for which there is no effective vaccine or antiviral treatment, and it is caused primarily by Coxsackievirus A6 (CVA-6) infection. Xie et al. (Xie et al., 2021) established a one-step reverse transcription RPA combined with disposable LFAs for the detection of CVA-6, which could be performed in less than 35 minutes at 37°C, with a sensitivity of 10 copies/reaction and a specificity of 100%. In the validation of clinical samples, the method showed a 100% agreement with qPCR results; therefore, it provides a simple and rapid method for the diagnosis of CVA-6 in clinical settings. The newly developed RPA-LFA can also be used for rapid and easy detection of the hepatitis B virus. Zhang et al. (Zhang et al., 2021) designed primers and probes targeting the conserved region of the hepatitis B virus and applied them to RPA-LFA, which was demonstrated to be able to detect hepatitis B virus of up to 10 copies/reaction by reacting under isothermal conditions at 39°C for 30 minutes, without cross-reactivity with other common pathogens. It is highly specific and sensitive and can be used as a screening test, providing an alternative method for hepatitis B virus detection in rural areas lacking equipment and making a meaningful breakthrough in the early monitoring, investigation, and control of hepatitis B virus infection.

### 4.2 Bacteria

The traditional bacterial detection method is mainly the isolation and culture of bacteria and their identification based on size, morphology, color, and hemolysis. However, because the culture of bacteria needs a certain environment and long culture time, it is unsuitable for the rapid detection of bacteria to manage some serious infectious diseases (Tan et al., 2022). Bhat et al. (Bhat et al., 2022) established a rapid and specific method to detect highly virulent Klebsiella pneumoniae. This RPA-LFA method introduced probes and base substitutions to eliminate false positive results, and it had good interspecies specificity and sensitivity, could be completed within 30 minutes at 37°C, and provided technical support for the rapid and accurate clinical identification of highly virulent Klebsiella pneumoniae. Yang (Yang et al., 2020) established a modified RPA-LFA method for the visual detection of Vibrio parahaemolyticus, which provided a rapid, accurate, and simple Vibrio parahaemolyticus detection method suitable for on-site detection under resource-limited conditions. In addition to Vibrio parahaemolyticus, Staphylococcus aureus, Enterobacteriaceae, O157:H7, and Lactobacillus monocytogenes are five common foodborne pathogens that cause various foodborne illnesses. Jin et al. (Jin et al., 2022) used a small automated nucleic acid extractor combined with RPA and RPA-LFA for simultaneous quantitative detection of five major foodborne pathogens, which allows for the rapid identification of the foodborne pathogen species responsible for food poisoning, leading to its timely and effective control. RPA-LFA can also be applied to detecting bacterial resistance genes. Lu et al. (Lu et al., 2023) established a RPA combined with LFA method for the simultaneous detection of highly prevalent drug resistance genes in Enterobacteriaceae pathogens, including Mcr-1, blaNDM-1, and tet (X4). The method can better detect and monitor a wide range of antibiotic multi-drug resistance genes in foodborne pathogens and rapidly detect resistance genes to identify the specific drugs that the pathogen is sensitive to, and thus aid the rapid control of diseases of great significance.

### 4.3 Fungi

Clinical infections caused by conditionally pathogenic fungi are becoming more severe due to modern lifestyles and increasing medical interventions, with Candida and Cryptococcus being the most common invasive fungi responsible for clinical fungal infections (Zhao et al., 2022). Most of the current diagnostic methods for fungi depend on traditional fungal isolation and culture to determine the diagnosis, but this method is time-consuming, taking approximately 48–72 hours. Furthermore, this method may lead to untimely intervention for the disease, increasing the risk of death; therefore, early detection of the causative fungi is of utmost importance. Cryptococcal meningitis is a subacute meningitis, and it is the most common cause of meningitis in adults. Moreover, its mortality rate is very high, and there is no widely used nucleic acid test for detecting cryptococci. Ma et al. (Ma et al., 2019). developed a new method for detecting cryptococcal genomic DNA using RPA-LFA to detect Cryptococcus neoformans in the cerebrospinal fluid. The entire process, from the preparation of the template to the test strip, required approximately 35 minutes; no special heating equipment was needed, and no professional staff was needed to interpret the results, saving time and human cost and making it an ideal method for the rapid and intuitive detection of Cryptococcus. Wang et al. (Wang et al., 2021). created a modified RPA-LFA system that eliminated the effect of primer dimers by using probes in the RPA reaction and introducing specific base substitutions in the primer and probe sequences, allowing for the specific detection of *Candida albicans* at 37°C in 25 minutes. Moreover, evaluation with the clinical isolates of *Candida albicans* and common causative organisms demonstrated that the method had good analytical specificity. The proportion of fluconazole-resistant non-Candida albicans infections, such as Candida krusei and Candida glabrata infections, is increasing every year. Zhao et al. (Zhao et al., 2022). designed two pairs of primers for RPA and qPCR based on the ITS2 sequences of *C. krusei* for the diagnosis of *C. krusei* infection and compared the sensitivity, specificity, and utility assays of RPA and qPCR techniques. The results showed that, as with the qPCR method, RPA had 100% specificity in diagnosing *C. krusei* infection, and the combination of RPA with LFA fulfilled the need for visualization and shortened the reaction time, which is beneficial to the early detection of *C. krusei* infection in resource-poor areas.

### 4.4 Parasites

Trichinella is one of the most widely distributed parasitic nematodes, living mainly in the muscles of many vertebrates and humans. Human infections are caused mainly by ingestion of raw or undercooked meat containing trichinella larvae, and the control and prevention of this parasite has been difficult due to the lack of effective parasite surveillance systems in many countries. Li et al. (Li et al., 2019b) established a simple, rapid, and accurate RPA-LFA for the detection of trichinella infections in domestic animals, which could detect trichinella DNA of as low as 100 fg with 10 times the sensitivity of the conventional PCR method, and amplification could be performed over a wide range of temperatures (25–45°C). Additionally, the amplification reaction could be completed within 10–25 minutes. This would be helpful for large-scale surveys of the prevalence of trichinellosis and has great importance for the effective control and monitoring of trichinellosis. Clonorchiasis sinensis is an important food-borne zoonotic parasitic disease caused by the consumption of freshwater fish containing infected cysticerci. Based on specific primers and probes, Ma et al. (Ma et al., 2023). established a new RPA method and combined it with lateral flow strips to achieve a rapid and intuitive assay for the detection of Clonorchiasis sinensis by amplification of the gene for the mitochondrial cytochrome C oxidase subunit 1 (COX1). Toxoplasmosis is a worldwide parasitic disease caused by the protozoan Toxoplasma gondii. Approximately one-third of the population is chronically infected with this pathogen, and infection with Toxoplasma gondii is usually asymptomatic in immunocompetent individuals but may cause fatal disease in immunocompromised patients. Furthermore, infection during pregnancy can lead to severe fetal damage, abortion, or fetal death. Wu et al. (Wu et al., 2017). established a method for the detection of Toxoplasma oocysts in soil and water by RPA coupled with lateral flow strips, which is sensitive, economical, and time-saving and provides a more practical technique for the detection of Toxoplasma DNA in the environment.

### 4.5 Other pathogens

Other pathogens, including spirochetes, mycoplasmas, rickettsiae, and chlamydia, can cause disease, in addition to common bacteria, fungi, viruses, and parasites. *Mycoplasma pneumoniae* (Mp) is a causative agent of pneumonia in humans, accounting for 20% of pneumonia cases. Mp infections are nonspecific and cannot be diagnosed solely on the basis of clinical manifestations and chest radiographs; therefore, the selection of a highly specific laboratory test is essential for the accurate diagnosis of Mp-induced diseases. Zhu et al. (Zhu et al., 2022a). used RPA combined with LFA for the diagnosis of Mp. The optimal reaction conditions for this method were a temperature of 37°C and a reaction time of 25 minutes, and it has a minimum detection limit of 10 DNA molecules with no cross-reactivity with 14 other common pathogens, providing a high degree of sensitivity and specificity. The RPA-LFA method established in this study could detect Mp in clinical samples.

## 5 Discussion

The many advantages of RPA-LFA make them the diagnostic tool of choice for the rapid, accurate, and cost-effective identification of various pathogens. RPA can be used to amplify 1–10 copies of a sample in less than 30 minutes under a low constant temperature, and the amplified product can then be added directly to a lateral flow strip without purification, following which the results can be read by the naked eye in 5–10 minutes. This is a simple procedure that does not require complex and sophisticated instrumentation or trained professionals. The whole process can be completed within 30–40 minutes, which greatly shortens the time needed for diagnosis. Increasingly, experiments are proving that RPA-LFA has sufficient sensitivity and specificity to meet the requirements of clinical pathogen detection.

Although RPA-LFA technology has been developed over a long period of time, it still faces many challenges. First, no software is available for designing specific RPA primers for RPA reactions, which may lead to inappropriate primer combinations, resulting in false positives. This reduces the sensitivity of the assay and makes establishing multiplex RPA systems difficult. To solve this problem, it is necessary to carefully consider the principles of RPA primer design, design multiple pairs of primers and probes for combinatorial screening, and determine the optimal reaction combination (Mota et al., 2022). In this regard, some studies have also successfully improved the specificity and sensitivity of the RPA reaction using strategies such as replacing the last four bases at the 3’ end of the primer with a self-avoiding molecular recognition system (SAMRS), which eliminates the interference by the primer dimer in the RPA reaction and promotes the amplification of the target gene (Sharma et al., 2014). Furthermore, Luo et al. (Luo et al., 2019) added cheap betaine to the RPA reaction, which significantly improved its specificity and efficiency. These methods enhanced the experimental performance of RPA and helped to develop applications in the field of RPA.

**Figure 5:**
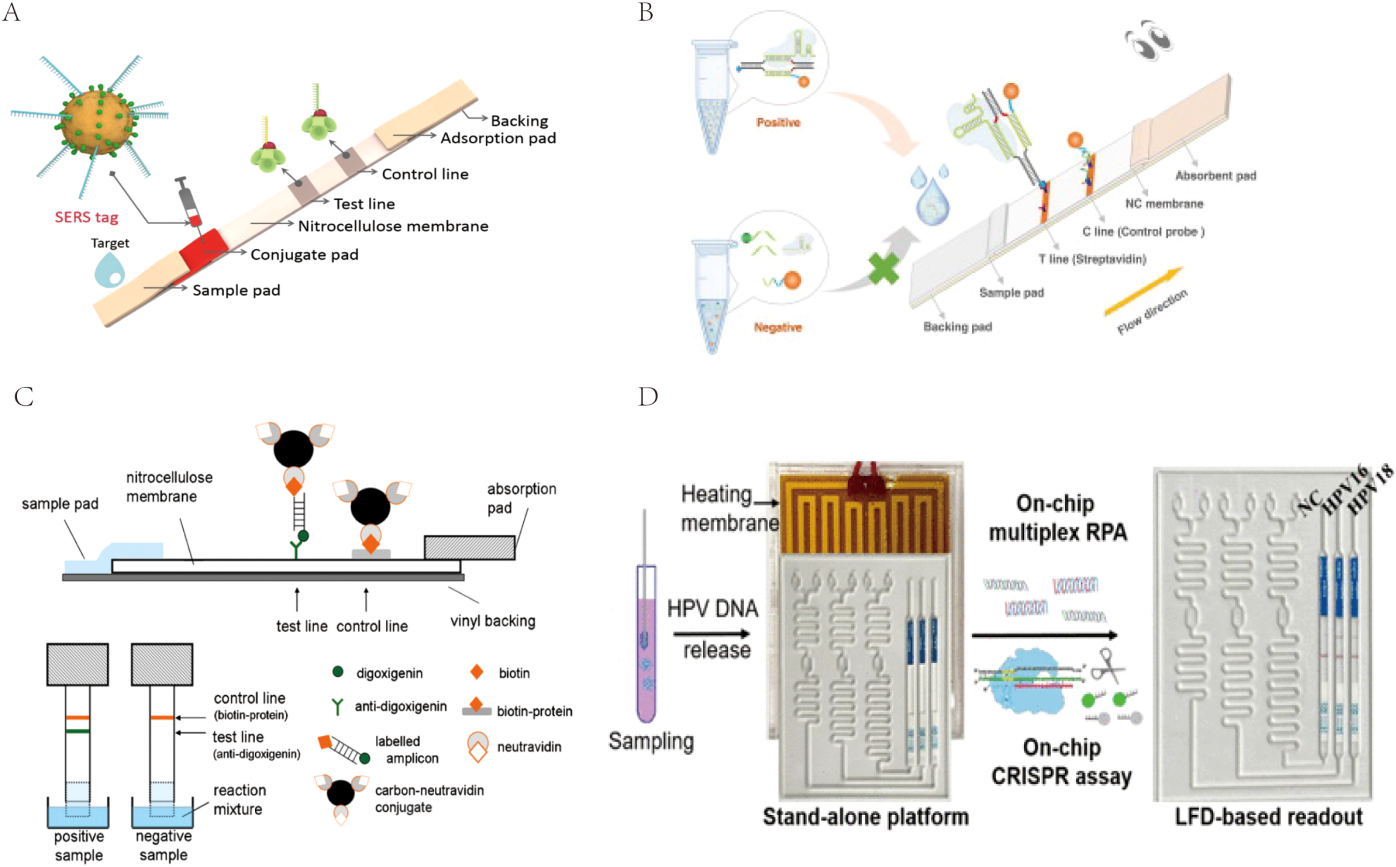
**(A)** The measurement principle of the SERS-based lateral flow assay for quantification of HIV-1 DNA. Reprinted with permission from (Fu et al., 2016), Copyright 2016 Elsevier. **(B)** Schematic illustration of CRISPR/Cas9-LFB for M. pneumoniae assay. Reprinted with permission from (Zhu et al., 2023) , Copyright 2023 Elsevier. **(C)** Schematic diagram of immunochromatographic strip test (ICT). Reprinted with permission from (Blažková et al., 2011), Copyright 2011 Elsevier. **(D)** Scheme of the coupling CRISPR/Cas12a and recombinase polymerase amplification on a stand-alone microfluidics detection platform. Reprinted with permission from (Zhou et al., 2023), Copyright 2023 American Chemical Society.

For the detection of RPA products, LFA is a cost-effective, time-saving, and simple-to-use method, but the traditional AuNP-based LFA has problems of relatively low sensitivity and a high false-positive rate that need to be overcome (Nan et al., 2023b). To address this issue, many researchers are applying various novel tag materials as well as new signal amplification strategies, such as surface-enhanced Raman scattering (SERS) (Hwang et al., 2016), CRISPR/Cas biosensing systems (Cao et al., 2022), novel nanoparticle substrates (e.g., colloidal palladium nanoparticles [PdNPs]), carbon nanoparticles (CNPs), gold magnetic nanoparticles, and quantum dots (QDs) (Zheng et al., 2021; Huang et al., 2016) to improve the inspection performance of this detection system. Fu et al. (Fu et al., 2016) proposed a new SERS-based LFA method for quantitative analysis of HIV-1 DNA in which Raman reporter-labeled AuNPs were used as SERS nanotags for targeting and detecting HIV-1 DNA instead of bare AuNPs in LF test strips (**Figure 5A**). By monitoring the DNA-coupled AuNPs with characteristic Raman peak intensities, HIV-1 DNA can be quantitatively analyzed with a high sensitivity of at least 1,000-fold higher than that of other methods. AuNPs integrated with SERS have increasingly gained attention due to their high sensitivity, especially with the development of portable SERS devices, promoting the development of SERS-AuNP-based LFA for POCT. CRISPR-Cas proteins are programmable genetic proteins that have nuclease activity that cleaves substrates indiscriminately by pairing with specific target sequences of CrRNAs, making them highly sensitive in nucleic acid detection. When combined with the CRISPR strategy, false positives can be effectively avoided via the dual variant detection of INA and sgRNA-guided activation processes, resulting in a significant increase in specificity and accuracy (Su, 2022). Moreover, many studies have demonstrated that the addition of a CRISPR-assisted step at the end of the RPA reaction can amplify amplicon signals, thereby further improving the LFA sensitivity and specificity (Arizti-Sanz et al., 2020; Broughton et al., 2020; Rivera et al., 2024). Liu et al. (Liu et al., 2023) established an RPA-CRISPR/Cas12a-LFA for the visual detection of *H. pylori* that was 100-fold more sensitive than conventional detection methods. Zhu et al. (Zhu et al., 2023). developed a CRISPR/Cas9-based POC LFA (CRISPR/Cas9-LFA) platform for the rapid visual detection of Mp, where CRISPR/Cas9 was used to specifically recognize target gene-induced amplification products after amplification, which made CRISPR/Cas9-LFA more robust in avoiding false positives (**Figure 5B**). CNPs have a high signal-to-noise ratio (black-to-white background) and excellent sensitivity; therefore, they can be visually inspected down to the picomolar range and are simple to prepare, highly stable, non-toxic, easy to couple, and do not require activation (Zheng et al., 2021). Blažková et al. (Blažková et al., 2011) used nucleic acid amplification combined with immunochromatographic principles to detect organisms of the genus Cronobacter, and colloidal CNPs were used as tracers on immunochromatographic test strips for visualization of the results (**Figure 5C**). The method can visually detect 10 ng of PCR product within 10 minutes, which greatly improves the sensitivity and detection speed. QDs are fluorescent semiconductor nanocrystals with the advantages of stability, broad adsorption, and strong luminescence, and they are considered to be the most promising reporter materials for the development of highly sensitive immunochromatographic methods (Huang et al., 2016). McMillan et al. (McMillan et al., 2016) developed a QD-based immunoassay to detect avian influenza A (H7N9) virus. Compared to the traditional hemagglutination test, the sensitivity of this method is nearly 16 times higher. The addition of these new technologies and materials gives the LF biosensor better sensitivity and specificity, making it perform well enough to ultimately provide users with better detection services.

Secondly, although directional LFAs can be visualized and read directly, this approach mostly relies on the subjective interpretation of the user and has limitations for quantitative analysis (Ince and Sezgintürk, 2022) (Boehringer and O’Farrell, 2021), which poses a challenge for accurate and standardized interpretation of test results. Therefore, the development of LFA integrated with reading systems is crucial for obtaining quantitative results in POC diagnostics. In response to this problem, many studies are being conducted to develop efficient, accurate, and cost-effective LF reading systems, and it has been demonstrated that, depending on the type of report, it is possible to use colorimetric, fluorescent, magnetic, photothermal, electrochemical, and electrophysiological methods, magnetic, photothermal, electrochemical, or dual-signal readers, but each method has its advantages and disadvantages (Park, 2022). However, simple e-reading devices with convenient and fast features are better suited to meet the needs of POC diagnosis, and the integration of imaging, data processing, storage, and communication features in smartphones equipped with high-resolution rear cameras makes their use as quantitative analysis tools for LFA possible (Jiang et al., 2019). Lee and Zang et al. used a smartphone system in conjunction with an LFA to achieve the quantitative analysis of aflatoxin B1 (Lee et al., 2013) and salivary cortisol concentration (Zangheri et al., 2015). With the current popularity of smartphones and their increasing functionality, this electronic device with image analysis can greatly compensate for the traditional limitations of LFA in quantitative analysis and is expected to become a powerful tool for quantitative analysis of LFA POCT.

However, the ideal POCT platform requires not only highly sensitive, specific, and accurate quantitative results but also easy, efficient, and comprehensive pathogen identification, ultimately achieving a user-friendly POC diagnostic modality. Chip-based microfluidic platforms are well suited to meet POCT needs, and microfluidic devices with integrated nucleic acid amplification and LFA will be a very promising area of research (Zheng et al., 2021). An independent microfluidic device can integrate multiple steps such as sample processing, amplification, and readout into one convenient device. At the same time, the setting of multiple independent reaction channels can prevent interference between reactions and also ensure the simultaneous detection of multiple pathogens. The user only needs to add the sample by simple operation and then wait for the result to be outputted, achieving real input-reply detection and efficient and comprehensive multi-pathogen detection without the need for operation by professionals. Li et al. (Li et al., 2022) developed a simple, sensitive, instrument-free, CRISPR-based diagnostic method for SARS-CoV-2 using a stand-alone microfluidic system (**Figure 5D**). The microfluidic chip integrates reverse transcription RPA, the CRISPR-Cas12a cleavage reaction, and lateral flow (LF) detection in a single, closed microfluidic platform to enable stand-alone, contamination-free visual inspection. Zhou et al. (Zhou et al., 2023) developed a multiplexed independent automated microfluidic platform for rapid and parallel detection of human papillomaviruses (HPV) subtypes. The system was optimized to integrate sample input, RPA detection, CRISPR detection, and visual readout of LFA on the microfluidic device, facilitating fast and convenient detection, reducing the possibility of contamination, and achieving simultaneous detection of HPV 16 and HPV 18. Microfluidics integrates multiple steps into a tiny chip, which significantly improves the detection efficiency of pathogen detection, maximizes compliance with the needs of POCT, and has now become the most promising research topic in the field of POCT (Wang et al., 2024).

Overall, RPA-LFAs are sensitive, specific, convenient, and low-cost, making them widely used in the diagnosis of common pathogens, such as viruses, fungi, bacteria, parasites, and other pathogens. However, inherent limitations hinder their use in clinical settings. At the same time, with the continuous development of signal amplification technology and the development and application of new labeling materials, the performance of the detection system has been further improved. Additionally, microfluidic systems that integrate nucleic acid extraction, amplification, and detection can further improve their detection efficiency, simplify cumbersome operations, and simultaneously promote high-throughput detection of pathogens. Finally, the increasingly powerful smartphone system can help the user to perform rapid and quantitative analysis of the results, making it a truly user-friendly detection method. We speculate that in the future, RPA-LFAs will develop towards increasing convenience and reliability, such as the development of microfluidic chip detection devices that integrate multiple reaction steps to achieve sample input and result output functions. Combined with the powerful smartphone application software, patients can analyze results without leaving their homes. Finally, we believe that with continuous research and performance improvement, RPA-LFA will become an ideal tool for POCT that will be widely used for the rapid diagnosis of pathogens, the primary screening of pathogens in resource-poor areas, and the ultimate improvement of global healthcare.
